# Relative contribution of sensory and motor impairments to mobility limitations in children with cerebral palsy: an observational study

**DOI:** 10.1038/s41598-023-30293-9

**Published:** 2023-02-24

**Authors:** Hsiu-Ching Chiu, Louise Ada, Rong-Ju Cherng, Chiehfeng Chen

**Affiliations:** 1 Department of Physical Therapy, I-Shou University, Kaohsiung, Taiwan, ROC; 2 Discipline of Physiotherapy, The University of Sydney, Lidcombe, Australia; 3 Institute of Allied Health Sciences and Department of Physical Therapy, College of Medicine, National Chung Kung University, Tainan, Taiwan, ROC; 4 School of Public Health, Taipei Medical University, Taipei, Taiwan, ROC

**Keywords:** Paediatric research, Neuronal development

## Abstract

The purpose of this study was to determine the relative contribution of sensory and motor impairments to mobility limitations in cerebral palsy. An observational study was carried out in 83 children with all types of cerebral palsy with a mean age of 10.8 years (SD 1.2). Five impairments (coordination, strength, spasticity, contracture, proprioception) and three aspects of mobility (standing up from a chair, short and long distance walking) were measured. Standard multiple regression was used to determine the relative contribution of impairments to mobility as well as the relative contribution of strength of individual muscle groups (dorsiflexors, plantarflexors, knee extensors, hip abductors and hip extensors) to mobility. Five impairments accounted for 48% of the variance in overall mobility (p < 0.001): coordination independently accounted for 9%, contracture for 4% and strength for 3% of the variance. Five muscle groups accounted for 53% of the variance in overall mobility (p < 0.001): hip extensors independently accounted for 9%, knee extensors for 4%, dorsiflexors for 4% and plantarflexors for 3% of the variance. Our findings demonstrate that the impairments making a significant independent contribution to mobility in pre-adolescent cerebral palsy were loss of coordination, loss of strength and contracture.

## Introduction

Cerebral palsy is a non-progressive neurological condition resulting in motor impairments, the severity of which can interfere with mobility over the lifespan. Although the condition is not unchanging, impairments of the neuromuscular, musculoskeletal, and sensory systems become features of early childhood. Impairments can be either primary impairments which are an immediate result of the existing pathology such as weakness or proprioception, or secondary impairments which develop over time such as contractures or skeletal malalignments^1^. These impairments can impact mobility such as walking^2^. Children undergo therapy throughout their childhood in order to achieve maximum mobility which reaches a plateau approaching the 6th birthday^1^. However, mobility can gradually decline after the 9th birthday^3^. Chiu et al.^4^ found that walking performance is not stable for all levels of severity from childhood to adulthood in cerebral palsy and it can change, especially Gross Motor Functional Classification (GMFCS) Level III.

Walking is the main method of moving around. An observational study of children with cerebral palsy revealed that children within GMFCS Level III participated less at school than those within GMFCS Level I or II^5^. Improvement in walking speed over time could reduce mortality because walking speed is referred to as the sixth vital sign^6^. Maintaining walking speed is important because it is related to recreation^7^. Children with better mobility are more likely to maintain friendships with peers and to participate fully in society as they move into adolescence^8^. Although walking has received the most attention, there are other aspects of mobility which are also important for everyday life, such as standing up from sitting. For example, the ability to stand up from a sitting position is essential for children with cerebral palsy to be at school independently (i.e., managing the toilet and changing classrooms). Most children with cerebral palsy have difficulty executing tasks that require a substantial effort close to their maximal capacity. Standing up from a seated position might be one such activity, since it has been shown that older adults use near maximal lower limb muscle strength to stand up from a chair. That is to say that any change in ability to stand up from a chair could be an early sign of decline in mobility. However, the role of this task has received little attention in children with cerebral palsy^9^.

Just as important is the fact that there has been little examination of the *relative* contribution of impairments underlying mobility limitations in older children. A group from Belgium investigating the association between impairments and walking and have recently concluded that “further research should investigate which impairments or combination thereof are associated with gait deviations”^2^. This lack of knowledge may be why a systematic review found limited evidence to support the effect of various interventions for improving walking speed in children with cerebral palsy^10^. We therefore set out to (1) compile a comprehensive profile of the sensory and motor impairments underlying mobility limitations across the spectrum of ambulation and (2) examine the *relative* contribution of impairments (i.e., contracture, spasticity, proprioception, coordination, muscle strength) to mobility limitations in pre-adolescent children with cerebral palsy. A better understanding of which impairments contribute to mobility in ambulatory children with cerebral palsy could enhance intervention by targeting specific impairments. Our hypothesis was that loss of coordination would make an independent contribution to mobility.

## Methods

### Design

A cross-sectional study was carried out in Taiwan. Pre-adolescent children with cerebral palsy were recruited from elementary schools via video advertising, social media or referral from school therapists and cerebral palsy liaison officers. A physiotherapist with 15 years of experience collected measures from all participants in one session of about an hour. Familiarization trials were performed for all measures. Ethical approval was obtained from the National Cheng Kung University Human Research Ethics Committee (Approval No: NCKU HREC-F-106-005-2, 2017). We confirmed that all methods were performed in accordance with the relevant guidelines and regulations. Informed consent was obtained from all participants and parents/guardian of participants prior to data collection.

### Participants

Children with cerebral palsy were eligible if they: had been diagnosed as spastic cerebral palsy, were at least 9 but less than 12 years old, and were able to walk with or without an assistive device. The first author screened if the potential participants were able to follow the instructions of the measures by showing a video and/or talking to teachers and parents. Children were excluded if they had severe cognitive deficits. Sampling was stratified based on walking speed. The aim was to recruit 20 participants in each category of walking speed (< 0.4, 0.4–0.8, 0.8–1.2, > 1.2 m/s) in order to ensure adequate representation across Gross Motor Function Classification System (GMFCS) levels^11^ and types of cerebral palsy in the final sample. Characteristics of participants were recorded (age, sex, BMI, gestation, type of cerebral palsy, education class, walking speed, functional classifications [Gross Motor Function Classification System, Manual Ability Classification System^12^, Communication Function Classification System^13^, Eating and Drinking Ability Classification System^14^, physiotherapy, botulinum toxin injection and surgery) in order to describe the sample.

### Measurement of impairments

Four motor impairments (contracture, spasticity, coordination, and strength) and one sensory impairment (proprioception) were measured. Both sides were measured and averaged for the analysis. Contracture and spasticity were measured at the ankle because the ankle is one of the most common sites of contracture and spasticity clinically^15^.

Contracture of the ankle plantarflexors was measured once as range of motion of passive ankle dorsiflexion and reported in degrees. The participant was seated on a chair with their feet on a sliding board, knees and ankles flexed to 90°, and a weight of 5 kg placed on top of the knee. The examiner slid the foot back until the heel lifted off the ground, which represents maximum dorsiflexion. The angle between the vertical and the lower leg (described by a line from the lateral malleolus to the head of the fibula) was measured^16^, so that the smaller the number, the greater the contracture.

Spasticity of the ankle plantarflexors was measured once using the Tardieu Scale reported as a score from 0 to 4, i.e., 0 as no spasticity, 1 as slight resistance with no clear catch, 2 as clear catch at a precise angle, 3 as fatigable clonus less than 10 s and 4 as infatigable clonus more than 10 s. Participants lay in supine and relaxed while the examiner moved the foot into dorsiflexion as fast as possible and rated the muscle reaction^17^.

Proprioception was measured using the lower-extremity matching task^18^ which measures position sense of the knee and reported in degrees. The participant sat with both knees at 90 degrees flexion with a vertical clear acrylic sheet inscribed with a protractor between their legs. They closed their eyes and the examiner moved one knee to 5 angles between 20° and 60° flexion randomly five times and the participant moved the other knee to align their ankles. The average discrepancy in degrees of knee range of motion was measured, so that the larger the number, the more impaired the proprioception.

Coordination was measured twice using the Lower Extremity Motor Coordination Test (LEMOCOT)^19^ and the best attempt reported in taps/s. Participants were seated on a chair with back support, without shoes, with feet resting on the floor, the heel on the proximal target, and the knee flexed as close as possible to 90°. Participants moved the foot as fast as possible between targets placed 20 cm apart for 20 s. Only accurate taps were counted.

Strength (dorsiflexors/plantarflexors, knee extensors and hip extensors/abductors) was measured during maximum voluntary isometric contraction in N using the PowerTrack II™ commander^20^ (Australasian Medical & Therapeutic Instruments P/L, Australia, 125 pounds rated capacity, linearity 1%). Position, stabilization and resistance was standardized for each muscle group. In supine, for four muscle groups, the hip and knee were flexed 90° with the lower leg supported. For hip abductors, the hip and knee were in neutral position. The lever arm was measured with a tape measure using bony landmarks (lateral malleolus, medial knee joint and greater trochanter) and the point of resistance. All muscle groups were measured twice and the best attempt was chosen for analysis. Maximum voluntary isometric contraction in N was multiplied by the length of lever arm to report Nm. For analysis, the sum was used as a single measure that reflected strength in the lower extremity.

### Measurement of mobility

Three aspects of mobility were measured (ability to stand up from a chair and walking speed over short and long distance) in order to encompass everyday situations.

Ability to stand up from a chair was measured once using the 5-Times-Sit-To-Stand Test^21^ reported in stands/s. Participants sat on an adjustable-height chair with hip flexed at 90°, knee flexed at 105° and feet flat on the floor. They then stood up and sat down five times as quickly as was safe. Timing began when the examiner said “Go” and stopped when the buttock touched the seat of the chair on the fifth repetition. Participants were allowed to use their arms for assistance when necessary.

Short-distance walking speed was measured once using the 10-m Walk Test^6^. The time taken to walk barefoot over 10 m was recorded at both preferred and fast speed and reported in m/s. The stopwatch started when a foot crossed the starting line and stopped when a foot crossed the end line. Participants walked 5 m before and after the start and end line to account for acceleration and deceleration.

Long-distance walking speed was measured once using the 6-min Walk Test. The distance walked in shoes in 6 min was recorded and reported in m/s. Other than standardized verbal encouragement^6^, no additional conversation between the evaluator and participant occurred. The evaluator stood near the participant throughout the test and recorded each 10 m lap.

The four mobility measures (5-Times-Sit-To-Stand Test, 10-m Walk Test with preferred and fast speed, 6-min Walk Test) were highly correlated (r ≥ 0.74, p < 0.001). For analysis, the average was used as a single measure that reflected mobility.

### Statistical analysis

Descriptive statistics were calculated for all participants and for each level of GMFCS and each type of cerebral palsy.

The recruitment of at least 75 participants provided enough statistical power of the independent variables since each independent variable (five sensory and motor impairments) required at least 15 participants in the regression analysis^22^. Simple linear regression was used to determine individual correlations between impairments and mobility as well as between the strength of individual muscle groups and mobility. We reported correlations as the Person’s Correlation Coefficient and set a significant correlation if *p* < 0.05. Standard multiple regression was used to determine the relative contribution of impairments (contracture, spasticity, proprioception, coordination and strength) to mobility as well as the relative contribution of strength of individual muscle groups (dorsiflexors, plantarflexors, knee extensors, hip abductors and hip extensors) to mobility. In order to ascertain the assumptions of multiple regression, normative distribution was inspected using normal probability plot of the residuals and the variance inflation factor was calculated for multicollinearity. Statistica, v.13.5 was used for all analyses^23^.

The children were very close in age so this was not a confounder but walking speed is known to be related to height, so the averaged mobility was adjusted by dividing height before the analyses presented in Tables 3 and 4.

## Results

### Characteristics of participants

Eighty-three children with cerebral palsy age ranging from 9 to 12 years old (mean 10.8 years, SD 1.2), of which 54 (65%) were male and 29 (35%) were female, participated in the study (Table 1). Fifty-five (66%) children were born prematurely, 24 (29%) had hemiplegia, 34 (41%) had diplegia and 25 (30%) had quadriplegia. All children could walk, 26 (31%) walked more than 1.2 m/s, 23 (28%) walked between 0.8 and 1.2 m/s, 15 (18%) walked between 0.4 and 0.8 m/s and 19 (23%) walked less than 0.4 m/s. Fifty-four (65%) still received physiotherapy services but only 1 h/wk week (SD 0.8). Forty-five (54%) had received botulinum toxin injection in the lower limb(s) and 21 (25%) had experienced surgery of the lower limb(s).

**Table 1:** Characteristics of participants.

Characteristic of participants	n = 83
Age (years), mean (SD)	10.8 (1.2)
Sex, n males (%)	54 (65)
BMI, mean (SD)	18 (3)
Gestation, n premature (%)	55 (66)
Type of cerebral palsy, n (%)	
Hemiplegia	24 (29)
Diplegia	34 (41)
Quadriplegia	25 (30)
Education class, n (%)	
Regular/resource	61 (73)
Special	22 (27)
Walking speed (m/s), n (%)	
< 0.4	19 (23)
0.4–0.8	15 (18)
0.8–1.2	23 (28)
> 1.2	26 (31)
GMFCS, n (%)	
Level I	25 (30)
Level II	32 (39)
Level III	11 (13)
Level IV	15 (18)
MACS (level), mean (SD)	2 (1)
CFCS (level), mean (SD)	2 (1)
EDACS (level), mean (SD)	1 (1)
Physiotherapy service, n (%)	54 (65)
Physiotherapy service (h/week), mean (SD)	1.3 (0.8)
Previous botulinum toxin injection in lower limbs, n (%)	45 (54)
Previous surgery in lower limbs, n (%)	21 (25)

*GMFCS* Gross Motor Function Classification System, *MACS* Manual Ability Classification System, *CFCS* Communication Function Classification System, *EDACS* Eating and Drinking Ability Classification System.

### Distribution of type of cerebral palsy, mobility and impairments by GMFCS level

**Figure 1:**
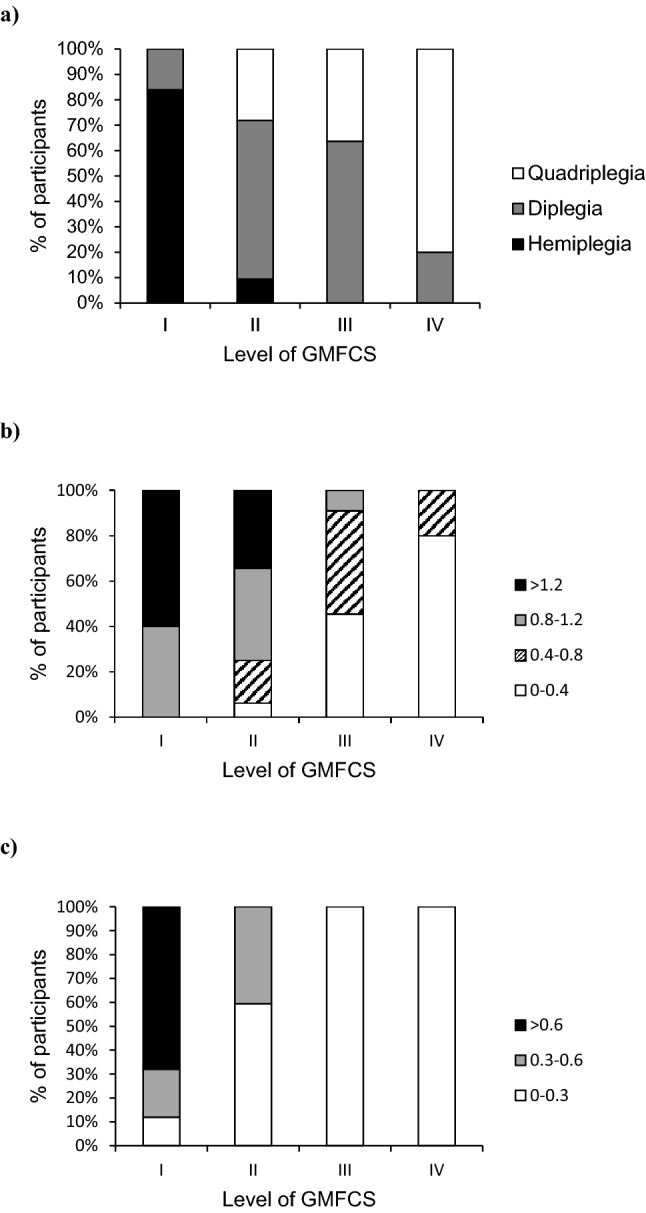
Distribution of (**a**) types of cerebral palsy, (**b**) walking speed (m/s) and (**c**) coordination (taps/s) across levels of Gross Motor Function Classification System (GMFCS).

Group data of mobility and impairments for all participants, by GMFCS level and by type of cerebral palsy are presented in Table 2 as well as reference values for typically developing children for 5-Times-Sit-To-Stand Test^21^, 10-m Walk Test with preferred and fast speed^24^, 6-min Walk Test^25^, strength^26^, coordination^27^, contracture^28^ and proprioception^29^. In terms of types of cerebral palsy, the majority of children with hemiplegia were within GMFCS Level I (21 out of 24, 88%), diplegia within GMFCS Level II (20 out of 34, 59%) and quadriplegia within GMFCS Level IV (12 out of 25, 48%) (Fig. 1a). In terms of mobility, all measures (5-Times-Sit-To-Stand, 10-m Walk test and 6-min Walk Test) decreased from GMFCS Level I to IV, e.g., walking speed decreased from 1.36 m/s (SD 0.40) for GMFCS Level I to 0.26 m/s (SD 0.21) for Level IV (Fig. 1b). In terms of impairments, two impairments (strength and coordination) decreased from GMFCS Level I to IV, one impairment (spasticity) increased from GMFCS Level I to IV and the other two impairments (contracture and proprioception) did not vary across GMFCS levels, e.g., coordination decreased from 0.76 taps/s (0.38) for GMFCS Level I to 0.04 taps/s (0.04) for Level IV (Fig. 1c).

**Table 2:** Mean (SD) of outcomes for all participants, each level of GMFCS and each type of cerebral palsy.

Outcome	All	GMFCS level				Type of plegia			Reference value
		I (n = 25)	II (n = 32)	III (n = 11)	IV (n = 15)	Hemi (n = 24)	Di (n = 34)	Quadri (n = 25)	
Mobility									
Standing up 5xSTS (stand/s)	0.32 (0.23)	0.55 (0.16)	0.34 (0.14)	0.17 (0.10)	0.02 (0.05)	0.55 (0.16)	0.31 (0.18)	0.11 (0.13)	0.57^21^
Walking speed (m/s)									
10-mWT-pref	0.93 (0.57)	1.36 (0.40)	1.07 (0.49)	0.48 (0.22)	0.26 (0.21)	1.42 (0.40)	0.85 (0.48)	0.58 (0.50)	1.21^24^
10-mWT-fast	1.32 (0.78)	1.93 (0.59)	1.53 (0.54)	0.62 (0.29)	0.34 (0.24)	1.86 (0.45)	1.24 (0.78)	0.89 (0.72)	1.83^24^
6-minWT	0.84 (0.46)	1.31 (0.27)	0.93 (0.22)	0.43 (0.17)	0.19 (0.11)	1.28 (0.31)	0.78 (0.38)	0.50 (0.38)	1.41^25^
Impairments									
Strength									
Handheld dynamometer (Nm)									
Dorsiflexors	4.6 (2.6)	6.2 (2.4)	5.2 (2.4)	2.7 (1.0)	1.8 (1.2)	6.4 (2.2)	4.3 (2.3)	3.2 (2.6)	23^26^
Plantarflexors	4.1 (2.9)	6.7 (2.1)	4.4 (2.1)	1.1 (0.7)	1.1 (0.9)	6.5 (2.3)	3.4 (2.5)	2.7 (2.5)	40^26^
Knee extensors	15.4 (10.4)	24.8 (8.8)	16.1 (7.4)	8.5 (4.7)	3.5 (3.4)	23.6 (8.2)	15.2 (9.3)	7.9 (7.5)	63^26^
Hip abductors	9.4 (4.8)	13.0 (4.0)	10.0 (4.3)	6.7 (2.9)	4.2 (1.5)	12.6 (4.0)	9.2 (4.8)	6.7 (3.8)	62^26^
Hip extensors	25.0 (9.0)	30.4 (7.6)	26.0 (8.5)	21.3 (5.5)	16.7 (7.6)	31.0 (7.7)	24.6 (7.9)	19.8 (8.5)	68^26^
Coordination LEMOCOT (tap/s)	0.36 (0.36)	0.76 (0.38)	0.30 (0.16)	0.08 (0.03)	0.04 (0.04)	0.69 (0.38)	0.32 (0.29)	0.09 (0.09)	2.36^27^
Spasticity Tardieu Scale (0–4)	1.3 (1.0)	1.0 (0.9)	1.3 (1.1)	1.4 (0.9)	1.7 (1.1)	0.9 (0.9)	1.5 (1.0)	1.3 (1.1)	
Contracture ROM of dorsiflexion (deg)	24 (10)	26 (9)	24 (10)	18 (13)	24 (12)	27 (7)	23 (11)	23 (12)	30^28^
↓Proprioception Lower extremity matching task (deg)	3 (2)	2 (2)	4 (2)	3 (2)	4 (1)	2 (2)	4 (2)	4 (1)	2^29^

*SD* Standard Deviation, *GMFCS* Gross Motor Function Classification System, *10-mWT* 10-m Walk Test, *6-minWT* 6-min Walk Test, *5xSTS* 5 times sit to stand, *OLST* One-Legged Stance Test, *LEMOCOT* Lower Extremity Motor Coordination Test.

### Relative contribution of impairments to mobility

Individually, contracture, spasticity, proprioception, coordination and strength were significantly correlated with mobility (Table 3). Together, the five impairments accounted for 48% of the variance in mobility adjusted for height (*p* < 0.001) (Table 4). While the shared component (correlation between impairments) accounted for 32% of the variance in mobility, three impairments (contracture, coordination, strength) were independently correlated with mobility. Coordination independently accounted for 9% (*p* < 0.001), contracture for 4% (*p* = 0.02) and strength for 3% (*p* = 0.04) of the variance in mobility.

**Table 3:** Relationship of impairments to mobility reported as Pearson’s Correlation Coefficients (r) and *p* value.

Impairments	Relationship to mobility	
	r	*p*
Contracture	0.23	0.03
Spasticity	− 0.23	0.04
Proprioception	− 0.29	0.01
Coordination	0.63	< 0.001
Strength (summed)	0.60	< 0.001
Dorsiflexors	0.61	< 0.001
Plantarflexors	0.63	< 0.001
Knee extensors	0.60	< 0.001
Hip abductors	0.52	< 0.001
Hip extensors	0.34	0.02

**Table 4:** Contribution of impairments and strength of individual muscle groups to mobility.

	Contribution to mobility	
	R^2^	*p*
Impairments		
Overall^a^	0.48	
Contracture	0.04	0.02
Spasticity	0.00	0.50
Proprioception	0.00	0.79
Coordination	0.09	< 0.001
Strength (summed)	0.03	0.04
Shared	0.32	
Strength of individual muscle groups		
Overall^b^	0.53	
Dorsiflexors	0.04	0.01
Plantarflexors	0.03	0.02
Knee extensors	0.04	0.01
Hip abductors	0.00	0.88
Hip extensors	0.09	< 0.001
Shared	0.33	

^a^Five impairments overall account for 48% of the variance in mobility.

^b^Five muscle groups overall account for 53% of the variance in mobility.

### Relative contribution of strength of individual muscle groups to mobility

Individually, dorsiflexors, plantarflexors, knee extensors, hip abductors and hip extensors were significantly correlated in mobility adjusted for height (Table 3). Together, the five muscle groups accounted for 53% of the variance in mobility adjusted for height (*p* < 0.001) (Table 4). While the shared component (correlation between muscles) accounted for 33% of the variance in mobility, four muscle groups (dorsiflexors, plantarflexors, knee extensors and hip extensors) were independently correlated with mobility. Hip extensors independently accounted for 9% (*p* < 0.001), knee extensors for 4% (*p* = 0.01), dorsiflexors for 4% (*p* = 0.01) and plantarflexors for 3% (*p* = 0.02) of the variance in mobility.

## Discussion

This study has compiled a profile of common mobility limitations and their underlying sensory and motor impairments in pre-adolescent ambulant children with cerebral palsy using a sample stratified by walking speed. In terms of severity of cerebral palsy, mobility declined across GMFCS Level I to IV and impairments increased, except for spasticity, contracture and proprioception which were similar across the levels. Individually, the five sensory and motor impairments (proprioception, strength, coordination, spasticity, and contracture) were all correlated with mobility. Relatively, coordination, strength and contracture made small independent contributions to mobility, although the shared component made the largest contribution, indicating interaction between these impairments. Likewise, individually, the five muscle groups (hip extensors/abductors, knee extensors, plantarflexors and dorsiflexors) were all correlated with mobility. Relatively, hip extensors, knee extensors, plantarflexors and dorsiflexors made small independent contributions to mobility although the shared component made the largest contribution.

It is interesting to compare the performance of our sample of ambulatory, pre-adolescent children with cerebral palsy to age-matched typically developing children. In terms of mobility, walking and standing up from a chair averaged about two-thirds of normal^18,21^. In terms of impairments, there was little spasticity, contracture or loss of proprioception. In contrast, coordination was only 15% of people without neurological conditions^26^ and strength also averaged only 15% of normal^26^ with the hip extensors being the strongest muscles.

In terms of severity and type of cerebral palsy, mobility declined sharply from GMFCS Level I to IV and from hemiplegia to quadriplegia, in particular the ability to stand up from a chair (which had largely disappeared by Level IV). Impairments increased from Level I to IV except for contracture and proprioception which were similar across severity and type. At one end of the spectrum, children within GMFCS Level I were equivalent to normal at most mobility tasks (112% at preferred walking speed, 105% at fast walking speed, 96% at standing up from a chair, 91% at long distance walking speed). However, strength and coordination were well below normal. Shortland^30^ suggests that these individuals are able to maintain good mobility because their muscle strength has not fallen below the threshold required to perform specific motor tasks.

Our findings are broadly in line with four previous studies investigating the relation between motor impairments and mobility^2,31–33^ in that individually, all impairments were related to mobility. However, relatively, both coordination and strength made independent contributions to mobility in our pre-adolescent children with cerebral palsy. This finding suggests that strengthening as an intervention could improve mobility. However, multiple systematic reviews^10,34–36^ have shown that any increase in strength of the lower limb muscles due to strength training does not carry over to improved walking. However, many of the trials included in these reviews are of mildly disabled children with cerebral palsy (Level I–II) whereas it may be children with more severe disability (Level III–IV) that would benefit. Shortland^30^ suggests that the importance of strengthening interventions may be to preserve muscle mass above a threshold required to perform a specific task. We know that muscle mass decreases over time in cerebral palsy and that this is accompanied by a reduction in motor function^37^. While maximum strength is not required for walking or standing up from a chair, this raises the question of what would happen if strength declined past a threshold level.

Perhaps it is more important to provide coordination training as a way of improving mobility, since we found coordination to be a major determinant of mobility, in line with previous studies^2,32^. Strength training with the requirement of coordination (i.e., high velocity and accuracy) in children with cerebral palsy may be more effective^38^. For example, two small trials found that high velocity contractions were effective in improving mobility in children^39^ and young adults^40^ with cerebral palsy. Furthermore, we know that gait training itself improves walking, presumably by improving the underlying coordination^10,38^. Lastly, the shared component made the largest contribution, implying the five impairments interact during mobility. Even though impairments such as proprioception or spasticity made no independent contribution to mobility, individually they were correlated with mobility.

Our finding that spasticity was related to mobility when examined individually is similar to previous studies^2,32,33^. However, relatively, we did not find that spasticity made an independent contribution to mobility. This is an interesting finding since spasticity is often clinically considered to be a major determinant of activity in spastic cerebral palsy. Lastly, we found contracture to be related to mobility individually in line with previous studies^2,32^. Furthermore, we found that relatively, it made an independent contribution to mobility. This suggests that preventing contracture may be an important goal of intervention.

This study has both strengths and limitations. One of its strengths is that it has a large, stratified sample. However, we were not able to recruit the same number into each stratum of walking speed and therefore we ended up with uneven cohorts within each level of severity. However, we did end up with similar numbers of each type of cerebral palsy. Another limitation is that we had to rely on previous publications for our normal values. This may introduce some inaccuracies given that (1) there are not many studies of normal which stratify by age, and (2) other studies may have used different methods to collect the data. It would be useful to collect normal values using the same method in a future study. In addition, the design of this study is cross-sectional, and we therefore do not know what changes in mobility will take place over time. It has been documented that the most common time for mobility to decrease to the extent that independent walking is lost is at adolescence^3,41^. Therefore, longitudinal tracking of impairments and mobility would be helpful to clarify how any change in impairments influence a change in mobility as children with cerebral palsy transition to adolescence.

## Data Availability

The datasets used and analysed during the current study available from the corresponding author on reasonable request.
